# Ambrisentan, an endothelin receptor type A-selective antagonist, inhibits cancer cell migration, invasion, and metastasis

**DOI:** 10.1038/s41598-020-72960-1

**Published:** 2020-09-28

**Authors:** Lucy Kappes, Ruba L. Amer, Sabine Sommerlatte, Ghada Bashir, Corinna Plattfaut, Frank Gieseler, Timo Gemoll, Hauke Busch, Abeer Altahrawi, Ashraf Al-Sbiei, Shoja M. Haneefa, Kholoud Arafat, Lena F. Schimke, Nadia El Khawanky, Kai Schulze-Forster, Harald Heidecke, Anja Kerstein-Staehle, Gabriele Marschner, Silke Pitann, Hans D. Ochs, Antje Mueller, Samir Attoub, Maria J. Fernandez-Cabezudo, Gabriela Riemekasten, Basel K. al-Ramadi, Otavio Cabral-Marques

**Affiliations:** 1 Department of Rheumatology and Clinical Immunology, University of Lübeck, Lübeck, Germany; 2 Department of Medical Microbiology and Immunology, College of Medicine and Health Sciences, United Arab Emirates University, Al Ain, United Arab Emirates; 3 Section Experimental Oncology, University Hospital and Medical School (UKSH), University of Lübeck, Lübeck, Germany; 4 Section for Translational Surgical Oncology and Biobanking, Department of Surgery, University of Lübeck and University Medical Center Schleswig-Holstein, Lübeck, Germany; 5 Lübeck Institute for Experimental Dermatology (LIED) and Institute of Cardiogenetics, University of Lübeck, Lübeck, Germany; 6 Department of Pathology, College of Medicine and Health Sciences, United Arab Emirates University, Al Ain, United Arab Emirates; 7 Department of Pharmacology and Therapeutics, College of Medicine and Health Sciences, United Arab Emirates University, Al Ain, United Arab Emirates; 8 Department of Hematology and Oncology, Faculty of Medicine, The University of Freiburg, Freiburg, Germany; 9 CellTrend GmbH, Luckenwalde, Brandenburg, Germany; 10 Department of Urology, Charité University Hospital, Berlin, Germany; 11 Department of Pediatrics, University of Washington School of Medicine, and Seattle Children’s Research Institute, Seattle, WA USA; 12 Department of Biochemistry, College of Medicine and Health Sciences, United Arab Emirates University, Al Ain, United Arab Emirates; 13 Department of Immunology, Institute of Biomedical Sciences, University of São Paulo, Lineu Prestes Avenue, 1730, São Paulo, SP Brazil; 14 Department of Clinical and Toxicological Analyses, School of Pharmaceutical Sciences, University of São Paulo, São Paulo, Brazil; 15 Network of Immunity in Infection, Malignancy, and Autoimmunity (NIIMA), Universal Scientific Education and Research Network (USERN), São Paulo, Brazil

**Keywords:** Drug development, Targeted therapies

## Abstract

Several studies reported a central role of the endothelin type A receptor (ETAR) in tumor progression leading to the formation of metastasis. Here, we investigated the in vitro and in vivo anti-tumor effects of the FDA-approved ETAR antagonist, Ambrisentan, which is currently used to treat patients with pulmonary arterial hypertension. In vitro, Ambrisentan inhibited both spontaneous and induced migration/invasion capacity of different tumor cells (COLO-357 metastatic pancreatic adenocarcinoma, OvCar3 ovarian carcinoma, MDA-MB-231 breast adenocarcinoma, and HL-60 promyelocytic leukemia). Whole transcriptome analysis using RNAseq indicated Ambrisentan’s inhibitory effects on the whole transcriptome of resting and PAR2-activated COLO-357 cells, which tended to normalize to an unstimulated profile. Finally, in a pre-clinical murine model of metastatic breast cancer, treatment with Ambrisentan was effective in decreasing metastasis into the lungs and liver. Importantly, this was associated with a significant enhancement in animal survival. Taken together, our work suggests a new therapeutic application for Ambrisentan in the treatment of cancer metastasis.

## Introduction

The endothelin type A receptor (ETAR) is a G-protein coupled receptor (GPCRs) expressed on both non-immune (e.g., endothelial cells, vascular smooth muscle cells, fibroblasts) and immune cells such as neutrophils^1–5^. ETAR, present on vascular smooth muscle cells, has a well-known role in vasoconstriction, which upon agonist stimulation by Endothelin-1 (ET-1), induces cell contraction^6,7^. Beyond that, ETAR exerts pleiotropic effects on the progression of ovarian, prostate, colon, breast, bladder and lung primary tumors and tumor metastasis^8–13^. The expression of ETAR on tumor cells increases migration, proliferation, and survival^14,15^. ETAR has also been reported to be overexpressed on breast cancer tissue in comparison with non-neoplastic tissue^10^ and tumor hypoxia induces breast carcinoma invasiveness in an ETAR-dependent manner^11^.

Currently, there is a unique selective ETAR antagonist approved for clinical use, which is called Ambrisentan (Volibris). Ambrisentan has been successfully explored to treat pulmonary arterial hypertension (PAH), and its safety has been demonstrated by several clinical trials^16–18^. However, Ambrisentan remains to be explored as a potential therapy for cancer. Here, we aim to investigate the potential role of Ambrisentan for the treatment of cancer. Since Ambrisentan is a selective ETAR antagonist it does not interfere with the physiological vasodilator and clearance effects of the endothelin type B receptor (ETBR)^19,20^. This counterbalances the ETAR effects by transducing intracellular signals that result in the production of nitric oxide and vascular relaxation^21^.

In the present study, we found that Ambrisentan inhibited tumor cell migration and invasion, while modulating the tumor transcriptome. Furthermore, in a pre-clinical breast cancer model, Ambrisentan reduced cancer cell metastasis to vital organs and decreased mortality. Taken together, our in vitro data using different tumor cell lines, and in vivo observations suggest a new application of Ambrisentan for the treatment of cancer.

## Results

### Ambrisentan demonstrates in vitro anti-tumor effects by impacting tumor cell migration and invasion

**Figure 1:**
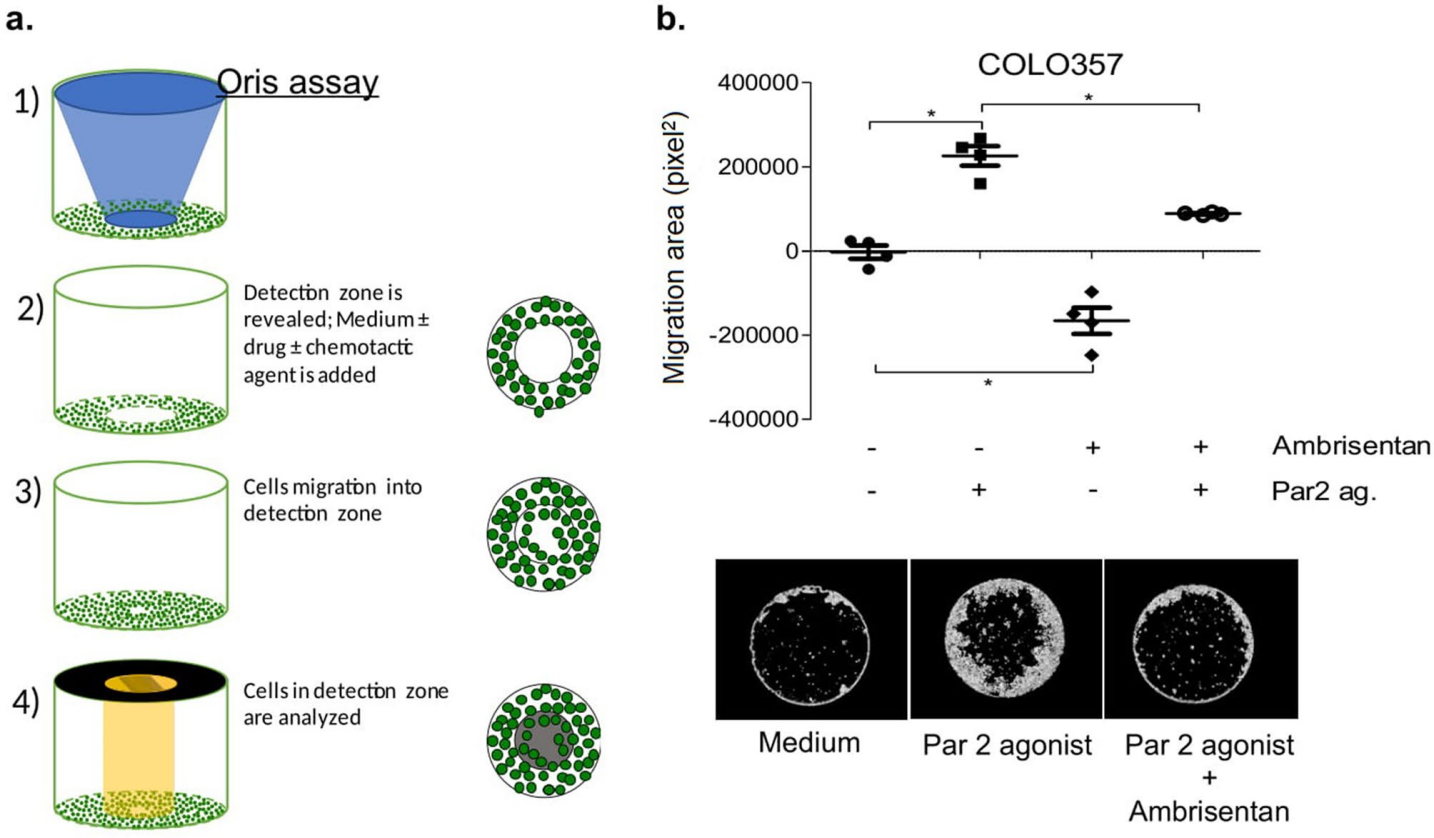
The anti-tumor actions of Ambrisentan on metastatic pancreatic adenocarcinoma (COLO-357) cells. (**a**) Illustration of the Oris migration assay (left panel), which was used to assess cell migration. (**b**) Graphics (on the top) and representative images (on the bottom) of the inhibitory effect of Ambrisentan on migration of COLO 357 cells before and after stimulation by PAR2 agonist (PAR2 ag.). Assays were performed in quadruplicates. Error bars denote mean with SD; *p ≤ 0.05 (n = 3, Mann–Whitney test).

**Figure 2:**
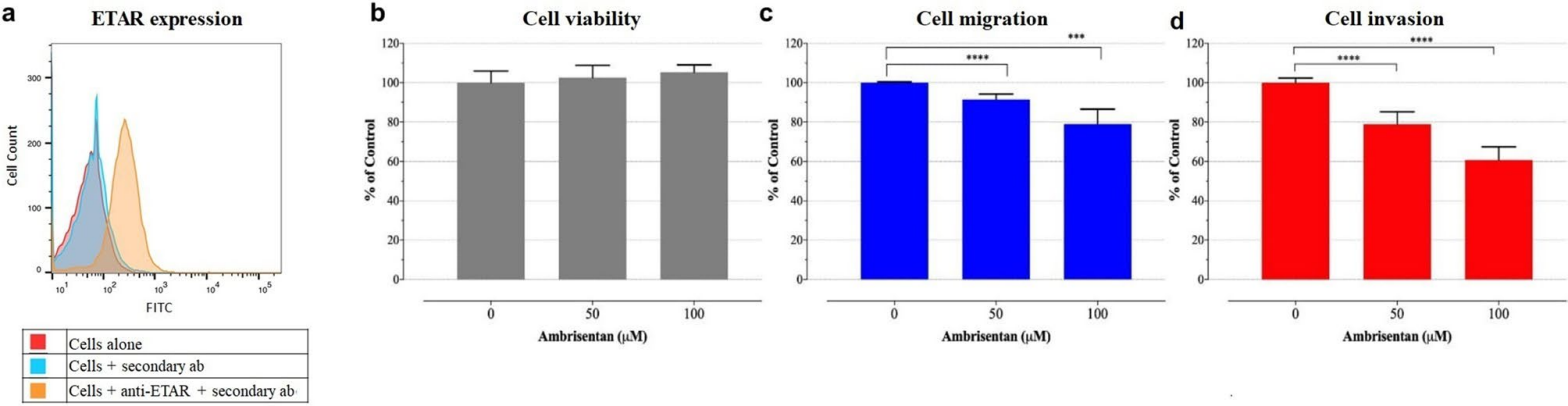
Ambrisentan inhibits migration and invasion capacity of MDA-MB-231 cells without any impact on cell viability. **(a)** Flow cytometry histogram shows the extent of ETAR expression by MDA-MB-231 cells. **(b)** Impact of Ambrisentan on cellular vaibility of exponentially growing MDA-MB-231 cells treated with either vehicle (0.1% DMSO) or the indicated concentrations of Ambrisentan for 24 h. **(c**, **d)** Viable cells that were able to cross the 8-mm pores insert (migration assay; **c**) and the matrigel matrix (invasion assay; **d**) were quantified using the CellTiter-Glo luminescent cell viability assay. The data are expressed as means ± SD of 2–3 replicates per group and are pooled from 3 independent experiments. Asterisks denote statistically significant differences between Ambrisentan-treated cells compared to controls (***p < 0.001; ****p < 0.0001).

In preliminary experiments, we tested the effect of exogenously added ET-1 on COLO-357 pancreatic carcinoma cells. However, the results showed no effect on migration (data not shown). It is well known that cancer cells secrete ET-1 constitutively. This could explain the lack of effect observed with exogenously added ET-1. We therefore assessed the effect of Ambrisentan on metastatic carcinoma cell lines in response to protease-activated receptor 2 (PAR2) activation, which is a GPCR that has been implicated in tumor progression^22–24^. For these experiments, we used the Oris Pro Cell assay, a technique employed to analyze the migration of adherent cells in real-time to a cell-free detection zone at the center of each well (Fig. 1a). Ambrisentan reduced the migration capacity of COLO-357 pancreatic carcinoma cells in the presence or absence of PAR2 induction (Fig. 1b). In concordance with these data, Ambrisentan also blocked the migration of other tumor cells such as OvCar3 ovarian carcinoma (Supplementary Fig. S1a) and HL-60 promyelocytic leukemia cell line (Supplementary Fig. S1b). We also analyzed the effect of Ambrisentan on the functional capacity of MDA‐MB231 breast adenocarcinoma, a cell line shown to be positive for ETAR expression (Fig. 2a). The findings demonstrate that treatment with up to 100 µM Ambrisentan had no effect on viability of cancer cells (Fig. 2b). However, the migration and invasion capacities of MDA-MB-231 cells, which are known to contribute to the tumorigenicity of these triple-negative breast cancer cells^25^ were significantly inhibited after a 24 h-exposure to Ambrisentan in a dose-dependent manner (Fig. 2c, d).

**Figure 3:**
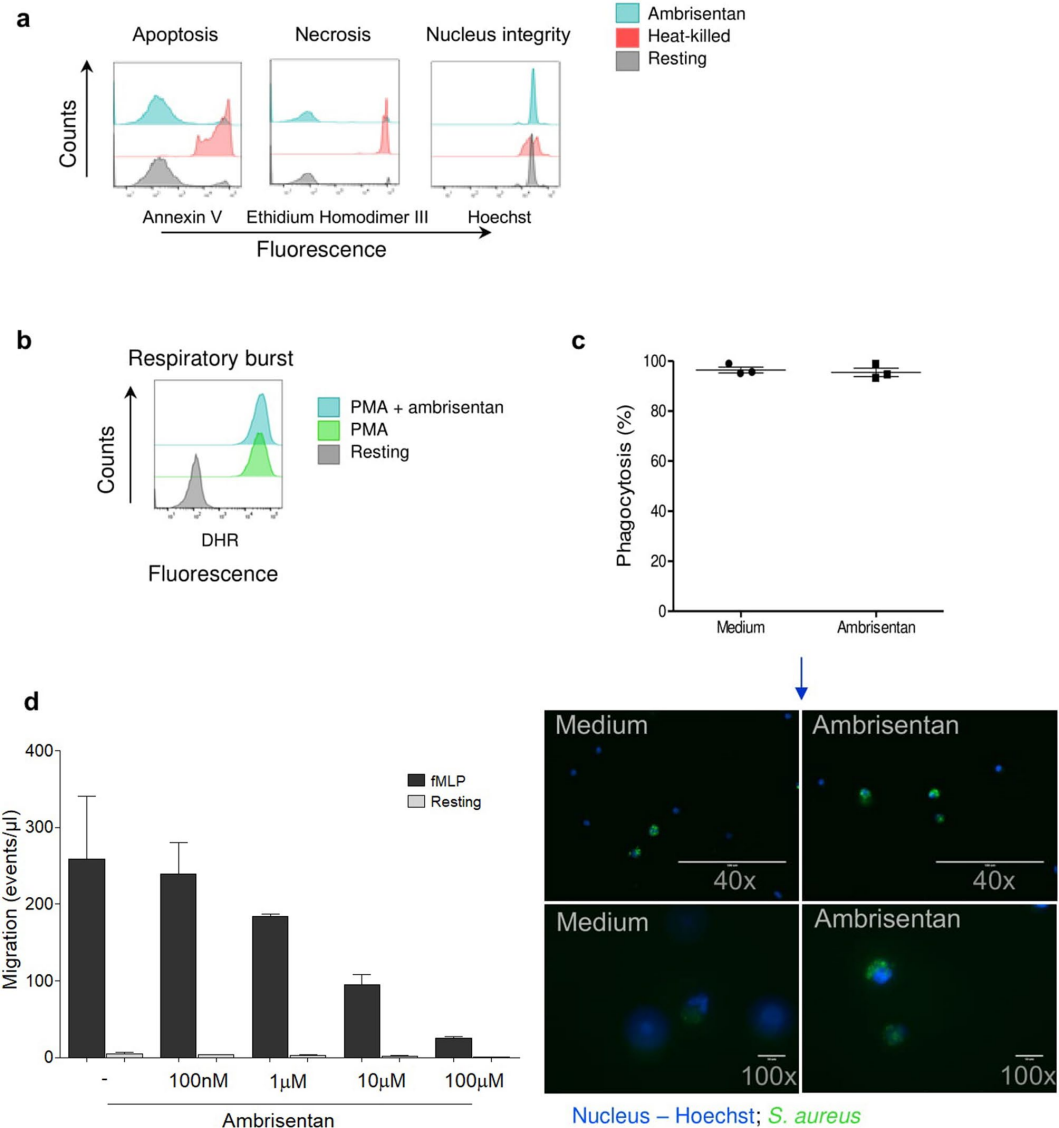
ETAR blockade inhibits migration of neutrophils while showing no cytotoxic effect. (**a**) Left histogram displays the apoptotic cells stained by FITC-annexin V; middle histogram shows the necrotic cells stained by ethidium homodimer-III; right histogram demonstrates healthy donor cells stained by Hoechst. Heat-killed cells were used as experimental control. (**b**) Neutrophil activation was evaluated in response to phorbol-12-myristate-13-acetate (PMA) by measuring the respiratory burst using dihydrorhodamine 123 (DHR 123). (**c**) Fluorescence images (lower panels using 40 × and 100 × objectives) and graphic (upper panel) of neutrophil phagocytic capacity, showing no effect of Ambrisentan on phagocytosis. Error bars denote mean with SEM; (n = 3; Mann–Whitney test). (**d**) Dose response-inhibition of neutrophil (from healthy subjects) migration by Ambrisentan.

Since high toxicity towards immune cells could indicate a limiting feature^26^ of ETAR blockade, we next assessed the effects of Ambrisentan on neutrophils from healthy donors. Neutrophils, which we recently showed to express ETAR^27^, are the most abundant peripheral blood-circulating leukocyte and have a very short lifespan^28,29^. Neutrophils exposed to Ambrisentan did not show apparent toxic effects as indicated by lack of cell apoptotic or necrotic signals while maintaining neutrophil nuclear integrity (Fig. 3a). In addition, Ambrisentan did not affect the respiratory burst response (Fig. 3b) or phagocytic capacity (Fig. 3c) of neutrophils, which are both essential for the development of protective immune responses. Despite these results, Ambrisentan significantly decreased neutrophil motility in a concentration-dependent manner regardless of whether a chemoattractant agent (fMLP) was present in the bottom of the transwell plates or not (Fig. 3d).

### Ambrisentan inhibits the activation of tumor transcriptome

**Figure 4:**
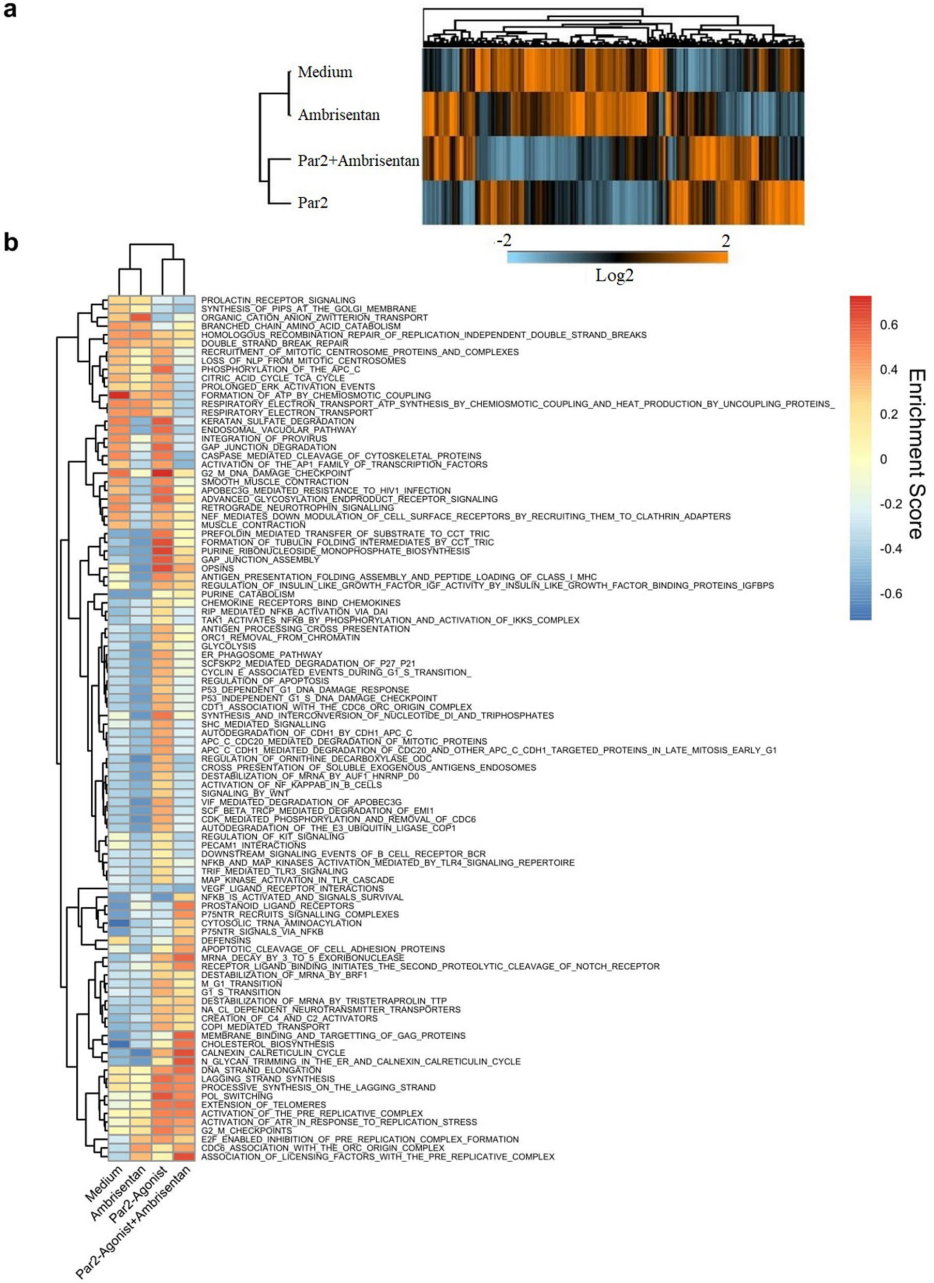
Effects of ETAR inhibition on the transcriptome of COLO- 357 cells. (**a**) Heatmap of hierarchical clustering shows the transcriptome profile of COLO-357 cells in the absence or presence of ambrisentan and/or Par2 antagonist. The transcriptional levels are represented in a log2 scale. (**b)** Gene Set Variation Analysis (GSVA) displays enriched (downregulated in blue and upregulated in orange) pathways in the absence or presence of ambrisentan and/or Par2 agonist.

**Figure 5:**
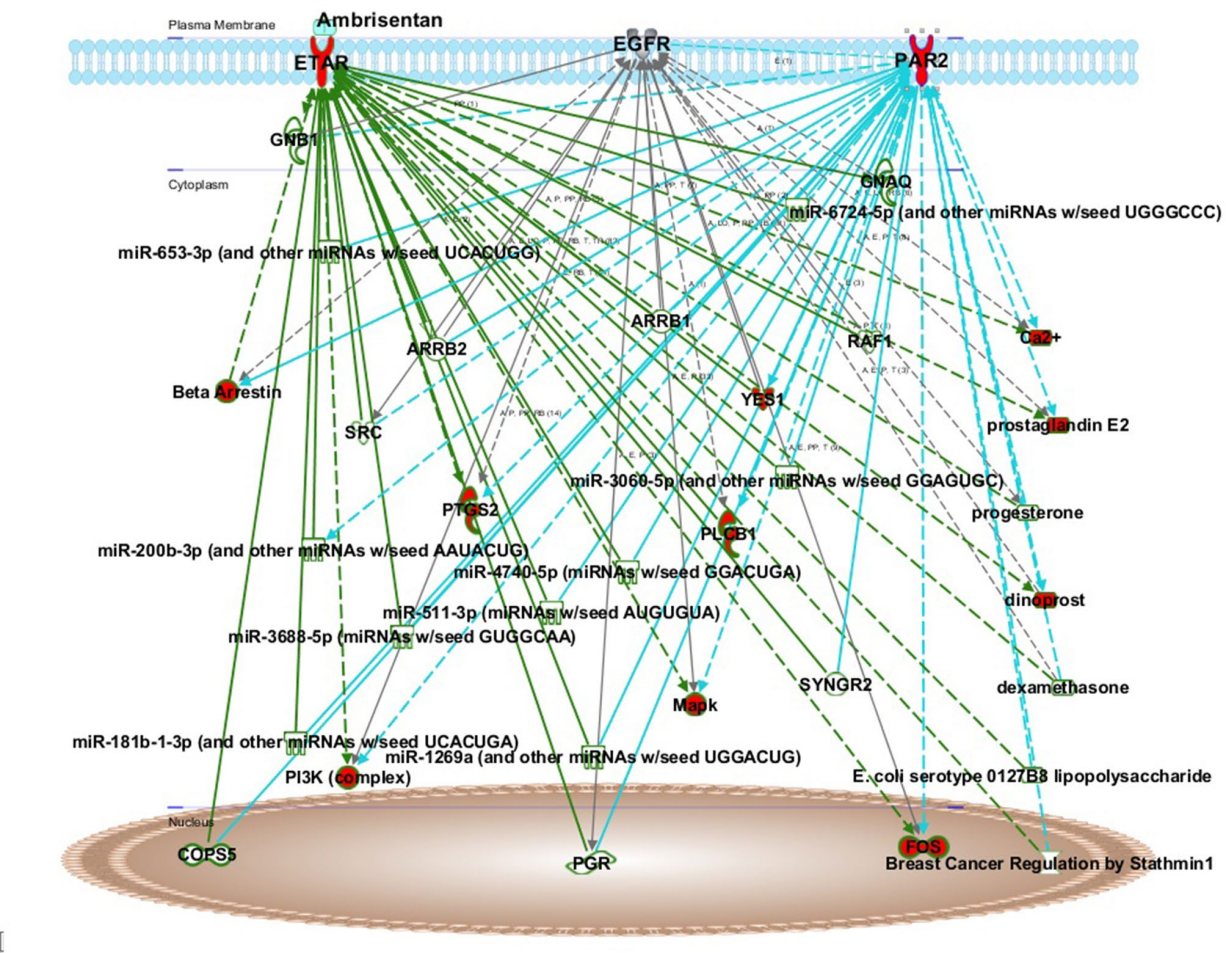
In silico analysis indicating an inhibitory effect of Ambrisentan on PAR2 signaling pathway. We applied QIAGEN’s Ingenuity Pathway Analysis (IPA, QIAGEN Redwood City, www.qiagen.com/ingenuity) tool to perform an in silico analysis of the effect of ETAR inhibition by Ambrisentan on PAR2 signaling. Molecules in red are predicted to be affected by Ambrisentan inhibition. Direct and indirect interactions are shown by solid and dashed lines, respectively.

Next, we evaluated the effects of ETAR blockade using Ambrisentan on the transcriptome profile of COLO-357 cells by RNA sequencing (RNA-seq). Hierarchical clustering (Fig. 4a) on the log_2_-transformed transcripts per million values (TPM) across all samples showed that Ambrisentan alone can change the transcriptome of COLO-357 cells. The transcriptome of COLO-357 cells treated with Ambrisentan plus the PAR2 agonist displayed a signature that was more similar with untreated (medium) cells when analyzed by hierarchical clustering compared to cells treated with PAR2 alone. In accordance, Gene Set Variation Analysis (GSVA) indicated that Ambrisentan alone is able to inhibit the activation of different reactome pathways that promote cancer initiation, progression, and metastasis (Fig. 4b) such as caspase-mediated cleavage of cytoskeleton proteins and activation of the AP1 family of transcript factors. Likewise, Ambrisentan reduced PAR2-induced activation of NF-κB and MAPK pathways as well as the formation of tubulin folding intermediates and chemokine receptor activity. Collectively, these data suggest multiple inhibitory effects of ETAR blockade on tumor-promoting signaling pathways. To test this possibility, we performed an Ingenuity Pathway Analysis (IPA) analysis which predicted an inhibitory effect of Ambrisentan on PAR2 signaling (Fig. 5). In addition, IPA analysis suggested the potential of crosstalk between ETAR and growth factor receptors, such as epidermal growth factor receptor (EGFR), as well as transactivation signaling events with other GPCRs. This is consistent with previous studies^30–34^.

### Ambrisentan inhibits tumor metastasis in vivo

**Figure 6:**
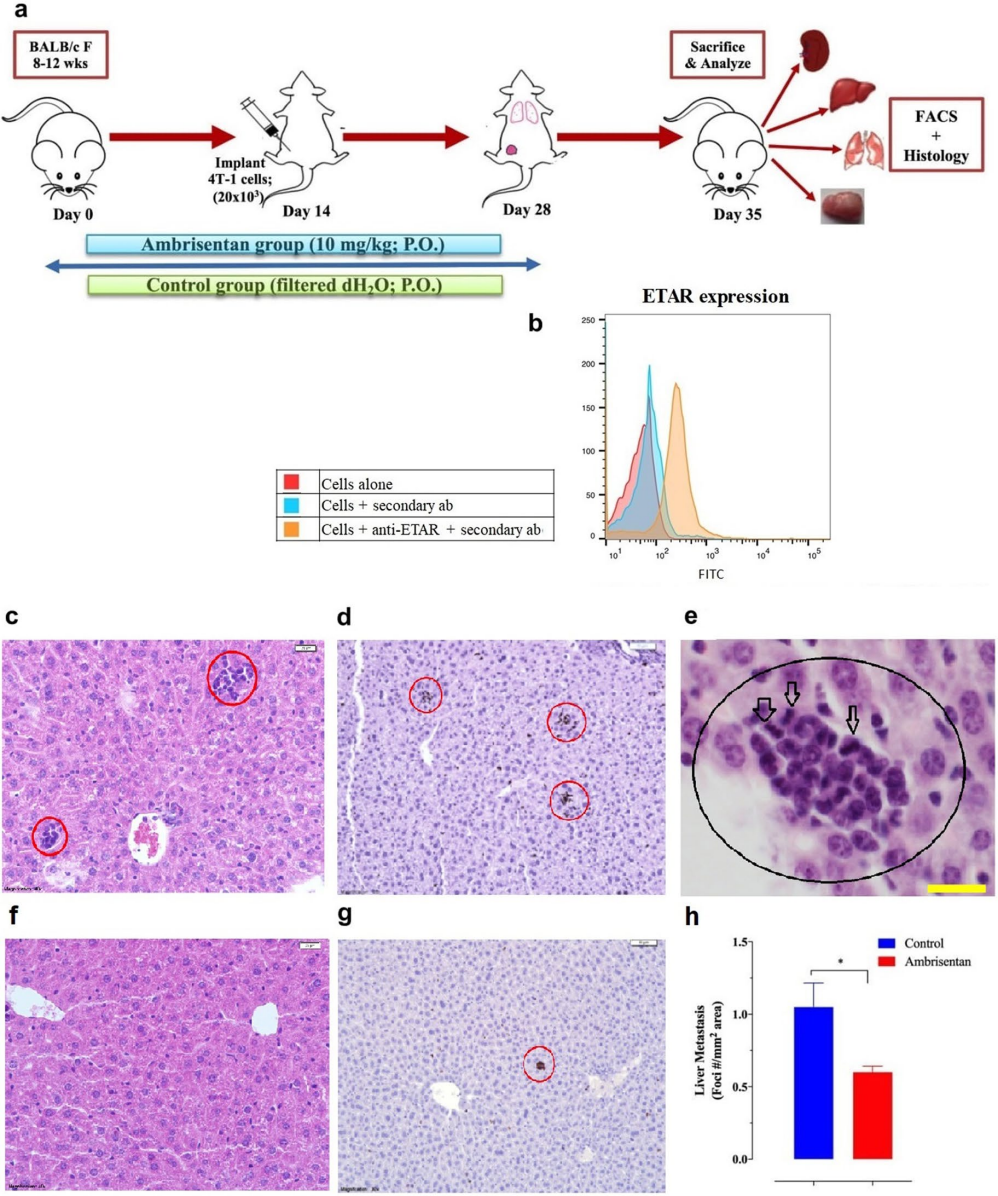
Assessment of liver metastasis in 4T1 breast tumor-bearing mice. (**a**) Schematic diagram of the treatment protocol for the orthotopic 4T1 breast cancer studies. Oral treatment with Ambrisentan was initiated 2 weeks pre-implantation of 4T1 cells and continued for another 2 weeks post implantation. Unless otherwise indicated, all animals were sacrificed on day 21 post implantation and organs/tissues were collected and processed for the indicated analysis. (**b**) Flow cytometry histogram shows ETAR expression on 4T1 cells. (**c-h**) Liver sections were prepared 3 weeks post implantation of 4T1 tumor cells and processed for H&E (**c, e, f**) and Ki67 (**d**, **g**) staining. Images taken at 40 × (**c, f**), 60 × (**e**) or 20 × (**d**, **g**) magnification are shown. Representative liver sections of control (**c-e**) or Ambrisentan-treated (**f**, **g**) mice are shown. Tumor metastatic foci consisting of a small cluster of tumor cells, strongly Ki67-positive, are circled in red. (**h**) The number of metastatic foci was determined for representative liver sections and calculated per mm^2^ area. Asterisks denote statistically significant differences (*p* < 0.05). The data is representative of 3 independent experiments (*n* = 11–12 / group). *ab* antibody.

Based on the data above, we further explored the anti-tumor effect of Ambrisentan on tumor growth and metastasis by evaluating its effect in a pre-clinical syngeneic mouse 4T1 breast cancer model. 4T1 cells are triple negative mammary gland tumors and, similar to human breast cancer, readily metastasize to the lungs, liver, and bone^35,36^. We assessed the effect of Ambrisentan administration on the extent of 4T1 metastasis by determining the number of metastatic foci in liver tissue sections. The details of the treatment protocol are shown in Fig. 6a. Flow cytometric analysis confirmed that 4T1 cells are positive for ETAR expression (Fig. 6b). We found that Ambrisentan-treated mice had significantly fewer metastatic foci (~ 43% reduction) compared to vehicle controls (Fig. 6c–h). These foci appeared clearly as small aggregates of tumor cells (Fig. 6c, e) and stained strongly with anti-Ki67 antibody (Fig. 6d, g), indicating a high proliferative state. The tumor foci had the characteristics of malignant cells including high nuclear cytoplasmic ratio, irregular contour, and abnormal mitotic figures (Fig. 6e). Furthermore, we compared these foci with the primary tumor for confirmation of their cellular characteristics (data not shown).

**Figure 7:**
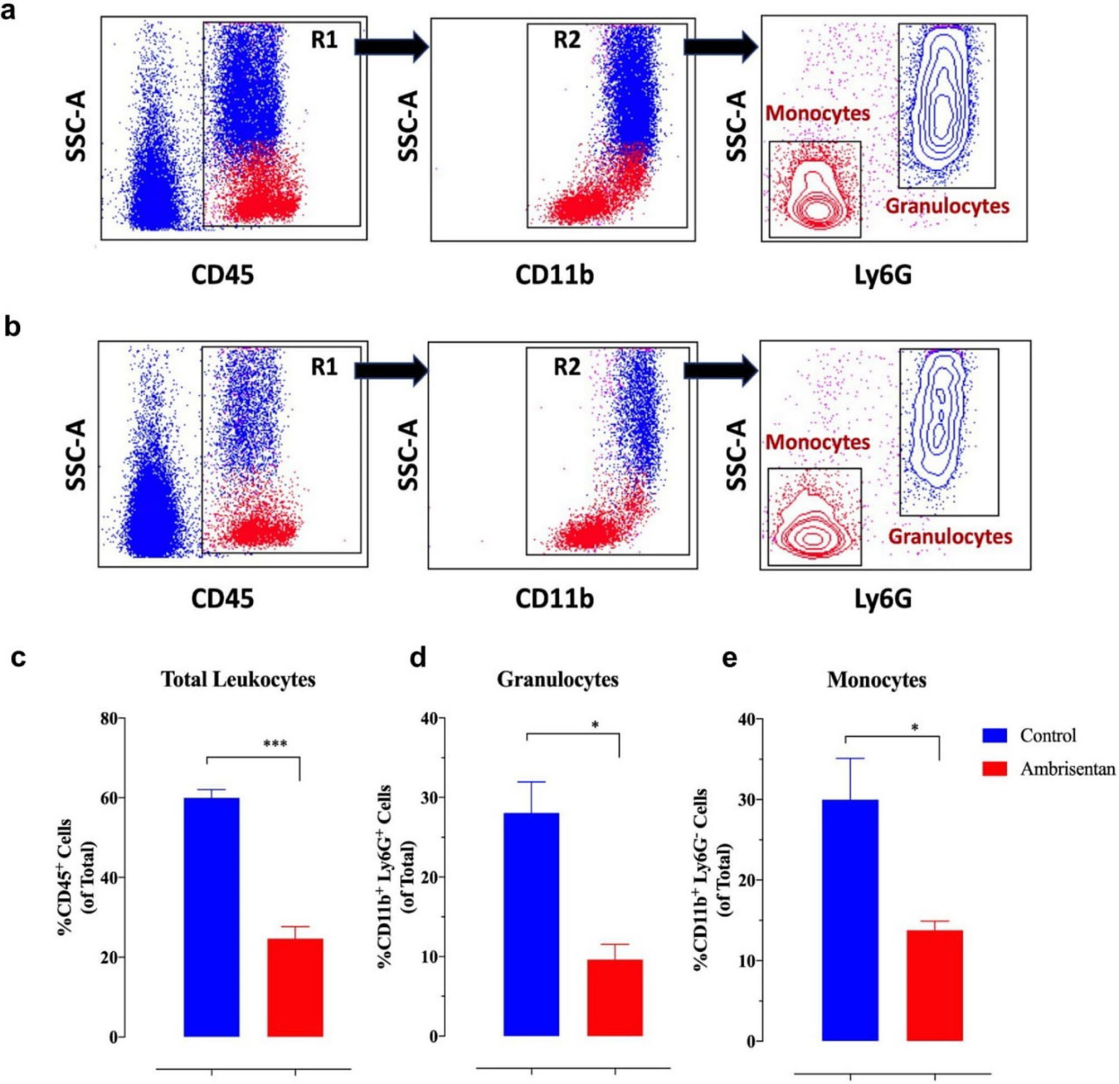
Flow cytometric analysis of mouse lungs following Ambrisentan treatment. (**a**, **b**) Dot and contour plots of gating strategy used for the identification of major myeloid cell populations in 4T1 tumor-bearing mouse lungs at day 21 post- implantation. Panels **a** and **b** illustrate representative flow plots from lungs of control or Ambrisentan-treated mice, respectively. Analysis was done after gating on viable cells (not shown). (**c**–**e**) Quantification of myeloid cell infiltration in the lungs of tumor-bearing mice. The percentages of CD45^+^ hematopoietic cells (**c**), CD11b^+^/Ly6G^+^ granulocytes (**d**), and CD11b^+^/ Ly6G^-^ monocytes (**e**) are shown. Asterisks denote statistically significant differences (*p* < 0.05). The data is representative of 2 independent experiments using 3 mice per group (*n* = 6).

By virtue of their high level of constitutive expression of monocyte chemoattractant protein-1 (MCP-1) and other chemokines^37^, 4T1 cells increased the accumulation of myeloid cells in the tumor tissue as well as in the blood and spleen of tumor-bearing mice^36^. Myeloid cell infiltration into the lung and liver of 4T1 tumor-bearing mice also occurred and correlated with cancer cell metastasis. Therefore, we used 6-color flow cytometry and sequential gating to analyze the extent of myeloid cell accumulation in the lungs of tumor-bearing mice with or without Ambrisentan pre-treatment. After the exclusion of doublets, debris, and non-viable cells, immune cells were identified using the pan-hematopoietic marker CD45 (Fig. 7a, b). Based on their positive expression of CD11b, most of the gated CD45^+^ cells in the lungs are of myeloid origin. These myeloid cells can be broadly subdivided into Ly6G-positive (granulocytes) or -negative (monocytes) populations (Fig. 7a, b). Pre-treatment with Ambrisentan reduced the accumulation of myeloid cells (66% and 54% for Ly6G^+^ and Ly6G^-^ populations, respectively) (Fig. 7c–e), reflecting decreased metastasis of 4T1 cells in the lungs.

### Ambrisentan improves host survival in orthotopically-implanted 4T1 model

**Figure 8:**
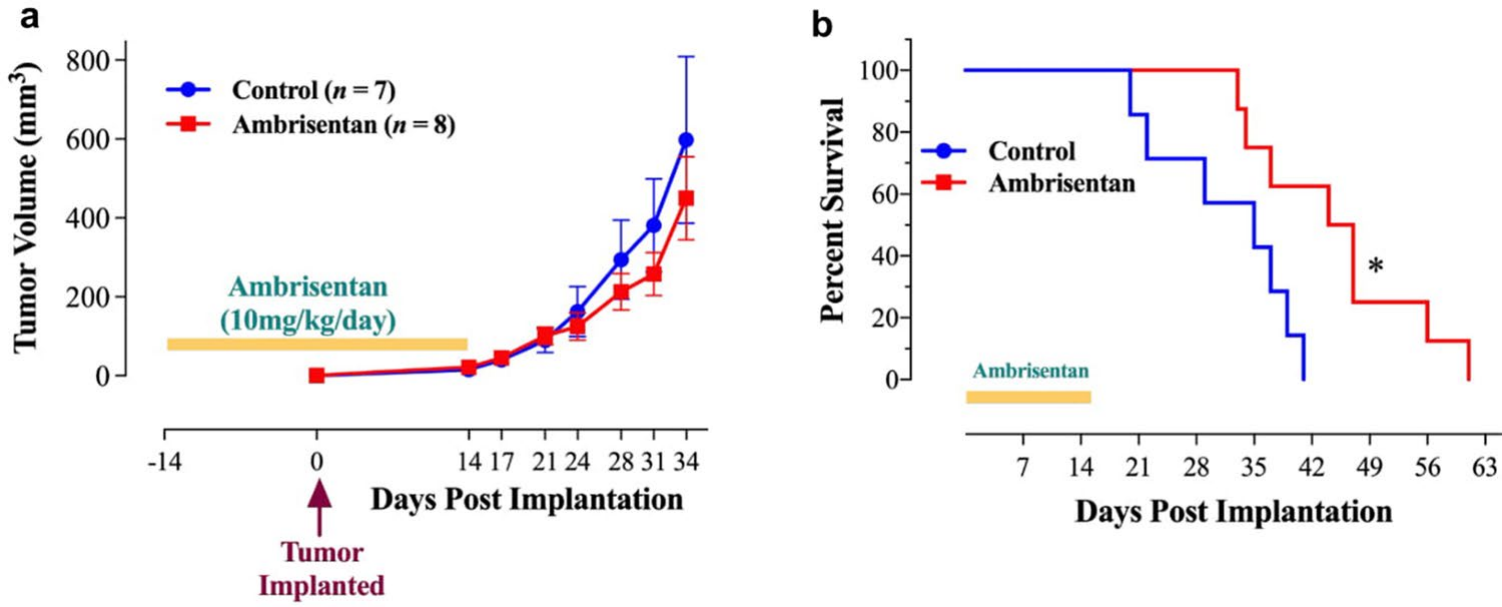
Increased mice survival after Ambrisentan treatment. Effect of Ambrisentan treatment on tumor growth (**a**) and host survival (**b**) after orthotopic implantation of 4T1 breast tumors. Ambrisentan was administered by daily oral gavage for 2 weeks pre-implantation and another 2 weeks post 4T1 tumor implantation. Animal survival was followed for up to 60 days. Numbers in parenthesis denote the number of mice per group. Asterisks denote statistically significant differences (*p* < 0.05). The data is representative of 2 independent experiments.

Next, we evaluated the effect of Ambrisentan administration (10 mg/kg/day) on tumor volume and host survival. While Ambrisentan demonstrated no reduction in local tumor growth within the mammary fat pad (Fig. 8a), the overall survival of tumor-bearing animals improved significantly (Fig. 8b). The median animal survival increased from 35 days in the control group to 45.5 days in Ambrisentan-treated mice (Fig. 8b). It is important to note that Ambrisentan (at doses up to 100 µM) had no effect on 4T1 cell proliferation in vitro (data not shown). These findings suggest that the anti-tumor effect of Ambrisentan appears not to be via inhibition of cell proliferation but, rather, primarily through its effect on cancer cell metastasis.

## Discussion

Drug repurposing represents an important pharmacological strategy for extending oncology therapies, and existing compounds originally developed for other purposes have the potential to be repurposed for cancer treatment^38,39^. The data reported herein suggests a potential therapeutic role of Ambrisentan in limiting the extent of cancer metastasis. Importantly, Ambrisentan has already been approved by the FDA and the European Medicines Agency (EMA) for the treatment of PAH^19,40–42^ and connective tissue diseases such as Systemic Sclerosis based on extensive safety, toxicity and efficacy testing^43^. Our findings are in agreement with recent reports showing that the dual-specific ETAR and ETBR antagonist, Macitentan, has the ability to counteract chronic lymphocytic leukemia cells by inhibiting in vitro migration and proliferation^44^, as well as tumor growth and metastasis in resistant ovarian cancer xenografts in mice^45^. Likewise, a recent report from Eun-Ju Im and colleagues found serendipitously that sulfisoxazole, which inhibits exosome secretion, limits the growth and metastasis of MDA-MB-231 and 4T1 cells by targeting ETAR^46^.

Cell migration, invasion and metastasis are complex mechanisms that involve the establishment and maintenance of cytoskeletal polarization and kinetics^47^ triggered by homo- and heterodimeric receptor cross-talk and cross communication between second messengers^48–51^. In this context, ETAR participates in a complex network of events between GPCRs and several growth factor receptors to regulate cell migration^27,52,53^. Our preliminary transcriptome data suggests that it is not a single and linear cellular mechanism that is affected by ETAR blockade, but rather more intricate systemic and dynamic signaling events that might be influenced by Ambrisentan treatment through divergent and convergent pathways involving GPCR-mediated transactivation signaling mechanisms^9,33,34,54^. Our transcriptome analysis is limited by the fact that we only used PAR2 to stimulate the cells and pooled the RNA obtained from each condition in three independently performed experiments. This precluded a more detailed interpretation and analysis to understand the network of the signaling complex affected by Ambrisentan.

ETAR mediates cellular processes by regulating several signaling pathways including NF-κB, HIF-1α, Akt, MAPK, Gαq/PKC, Src, EGFR, and GSK in an autocrine or paracrine fashion^33,55–58^, which are pathways involved in the development of cancer^59–66^. In fact, the results of our IPA analysis are consistent with these previous studies, which suggest the possibility that Ambrisentan’s inhibitory effects could also involve other tumor-promoting signalling pathways (e.g. EGFR) and transactivation signalling events^30–34^. Such possibility could explain the inhibitory effect of Ambrisentan in response to PAR2 agonist. In this context, additional experiments based on ETBR or ETAR silencing could provide insightful mechanisms on how the blockade of ETAR/ETBR might affect cancer metastasis. The effect of dual ET-1 receptor antagonists, such as Bosentan or Macitentan, in comparison to Ambrisentan, could also allow a better understanding of whether ETBR participates in the observed effects**.**

Although Ambrisentan is a highly selective ETAR antagonist, its selectivity varies considerably according to the dose, cell model, species (animal or human), and receptor systems^67,68^. Therefore, future studies of the effect of Ambrisentan on different cancer cells will need to address its binding affinity to ETAR depending on the type of tumor investigated. Even though the concentration of Ambrisentan that we used here is in line with other studies assessing endothelin receptor antagonists (ERAs) in toxicity assays with glioblastoma and breast cancer cells, it is very likely that at high concentration off-target effects (ETBR and EGFR)^69,70^ are also involved in the in vitro anti-tumor effects of Ambrisentan. It is important to emphasize, however, that our conclusions regarding the effect of Ambrisentan on cancer metastasis are primarily based on the findings of the in vivo breast cancer model.

In our study, we assessed the effectiveness of Ambrisentan in the murine breast cancer model using drug concentrations (5–10 mg/kg) recommended for the treatment of patients with PAH^17,41^. Pharmacologically, taking into consideration the known species-specific differences in metabolic activity between mice and humans, the doses we used are at least one log below what the equivalent doses for mice would have been. In other words, a dose of 10 mg/kg in humans is equivalent to about 123 mg/kg in mice^71^. Consequently, the fact that we demonstrated anti-tumor effects of Ambrisentan at 10 mg/kg concentration in mice highlights its potential for in vivo treatment. If one were to extrapolate from the findings of the murine model, we would predict that Ambrisentan’s anti-tumor activity in humans could be achieved with a much lower drug dose than currently used to treat PAH. At this stage, it will also be important to investigate whether new therapeutic approaches such as local drug delivery systems could improve the anti-tumor potential of Ambrisentan.

There is strong evidence of aberrant activation of ETAR in the development of cancer^9,15,46,72–75^. For instance, ETAR expression levels were found to be higher in breast cancer specimens compared with non-neoplastic breast tissue^10^. Additionally, tumor hypoxia has been shown to increase breast carcinoma invasiveness by releasing intracellularly stored ET-1^11^. ET-1 acting via ETAR is a growth factor for colorectal cancer cells^13^ and ETAR activation of epithelial cancer cells, cancer-associated fibroblasts, and endothelial cells contributes to colorectal cancer growth and neovascularization^8^. However, earlier trials using endothelin receptor antagonist (ERAs) were not successful even though they showed an anti-tumor effect pre-clinically. The reasons for this failure might be that these drugs have been used as a monotherapeutic approach or in combination with standard chemotherapy for patients with established metastatic disease^9,72,76^, suggesting that ERAs have no effect on advanced tumors. Therefore, new therapeutic strategies need to explore the potential of Ambrisentan and other ERAs as adjuvant drug^77^ for patients with non-metastatic disease to prevent metastasis^9,72^. Since Ambrisentan inhibited tumor metastasis in our orthotopic 4T1 breast cancer model, oral or local application of Ambrisentan for patients diagnosed with early stage tumor that has not yet spread to nearby tissues could improve the sensitivity of tumor cells to chemotherapeutic treatment. This would reduce the time course of therapy to which patients are currently submitted. A similar approach could also reduce the dosage of chemotherapeutic agents required for the treatment of aggressive types of tumors, including those that present with high metastasis and relapsing rates, such as breast cancer^77–79^.

Finally, further work will be required to address the potential role of Ambrisentan in the treatment of cancer and to better understand the mechanisms how this drug reduces metastasis. For instance, it is necessary to investigate whether Ambrisentan promotes cancer apoptosis. Studies using starved cells to evaluate the effect of Ambrisentan on proliferation, migration, and invasion of synchronized cell cycle phase could also be insightful. Ultimately, experiments analysing cell migration and invasion using complementary approaches such as live-cell imaging will provide a more comprehensive analysis of how Ambrisentan affects biological processes and metastasis. Taken together, our data suggest a novel therapeutic role for Ambrisentan for the treatment of various metastasizing tumors.

## Methods

### Ambrisentan for in vitro assays

Ambrisentan was obtained from Gilead Sciences (Foster City, CA). For all in vitro assays, except for the cancer cell viability assay with MDA-MB-231 cells in which Ambrisentan was dissolved in 0.1% DMSO, Ambrisentan was resuspended in RPMI (10 mM stock solutions, pH 7.0), stored at -20 °C until use, and Ambrisentan working solution (100 µM final concentration) obtained by dissolving the stock solution in RPMI medium.

### Cancer cell lines and culture conditions

The inhibitory effect of Ambrisentan was analyzed using cancer cell lines: COLO-357 of metastatic pancreatic adenocarcinoma, OvCar3 ovarian carcinoma, HL-60, and MDA‐MB231 breast adenocarcinoma cell lines. The HL-60 promyelocytic leukemia cell line was cultured for 6 days in the presence of 1% dimethyl sulfoxide (DMSO) as previously described^80^. After 6 days of incubation, cells were harvested, counted, and their viability determined by Trypan blue exclusion before performing migration assays. All cell lines constitutively express ETAR and ETBR (Supplementary Fig. S2). They were cultured in 10% panexin NTA Serum (Pan-Biotech, Aidenbach, Germany) or 10% fetal bovine serum (FBS) + DMEM high glucose medium (Gibco-ThermoFisher Scientific, Waltham, MA, USA) as previously described^81^. The mouse mammary gland tumor cell line 4T1^36^ was generously provided by Dr Jo Van Ginderachter (Vrije Universiteit Brussel, Belgium). Cells were grown in DMEM supplemented with 10% FBS (Gibco-ThermoFisher Scientific). To prepare cells for implantation, 4T1 cells were trypsinized, washed, resuspended in PBS to a single cell suspension, counted, and injected subcutaneously within 30 min.

### ETAR and ETBR expression

ETAR and ETBR expression was evaluated by quantitative real-time PCR (qPCR) as previously described^82^. RNA extraction of tumor cell lines (COLO357, OvCar3, MDA-MB231 and HL-60) was performed using NucleoSpin RNA Kit (Macherey–Nagel GmbH & Co. KG, Düren, Germany) according to the manufacturer’s instruction. RNA integrity was checked on a 1% agarose/TRIS–acetate-EDTA gel supplemented with GelRed Nucleic Acid Gel Stain (Biotium, Fremont, CA, USA). 1 µg of total RNA was transcribed into cDNA using iScript cDNA Synthesis Kit (Bio-Rad Laboratories, Inc., Hercules, CA, USA) according to the manufacturer’s instruction. qPCR was done using SsoAdvanced SYBR Green supermix (Bio-Rad Laboratories, Inc., Hercules, CA, USA) and run on the Bio-Rad CFX Connect Real-Time PCR System according to the manufacturer’s instruction. Specific primers and qPCR conditions are available upon request.

### ETAR expression by flow cytometry

In addition to the analysis of ETAR and ETBR by real-time PCR (Supplementary Fig. 2), the expression of ETAR by MDA-MB-231 and 4T1 cells was confirmed at the protein level by flow cytometry using a modification of a previously published protocol^83^. Briefly, 3 × 10^5^ cells suspended in staining buffer (PBS/1% FCS/ 0.1% NaN3) were pre-incubated with either Human Trustain Fcx or anti-mouse CD16/32 mAb (both from Biolegend, San Diego, CA, USA) to block FcγIII/II receptor sites on MDA-MB-231 or 4T1 cells, respectively. The cells were then stained with Zombie Aqua Viability dye (Biolegend). After washing, the cells were stained with ETAR-specific rabbit polyclonal antibody (Cat# MBS9203839; MyBiosource, San Diego, CA) for 30 min. After 3 rounds of washing in staining buffer, cells were incubated with FITC-conjugated goat anti-rabbit IgG secondary antibody (cat# Ab97050; Abcam, Cambridge, UK). Data were collected on 10,000 cells per sample using FACS Celesta (BD) and viable cells were analyzed using FlowJo software (BD Bioscience).

### Ambrisentan and cancer cell viability assay

The effect of Ambrisentan on cancer cell viability was carried out using MDA-MB-231 cells, as detailed previously^25^. Briefly, MDA-MB-231 cells (5 × 10^3^ cells/well) were seeded overnight in 96-well plate and then cultured in triplicate for 24 h with Ambrisentan dissolved in 0.1% DMSO. Control cultures were treated with 0.1% DMSO alone (the drug vehicle). Cell viability was determined after 24hrs incubation the CellTiter-Glo Luminescent Cell Viability Assay (Promega Corporation, Madison, WI), based on quantification of ATP, which signals the presence of metabolically active cells. Luminescence was measured using a Glomax Luminometer (Promega) and normalized to control. The data are presented as percent cell viability of experimental groups compared to that of control cells. If indicated, the inhibitory effect of Ambrisentan (100 µM) was tested after 1 h of incubation with different concentrations of drug.

### Cell migration and invasion assays

The invasiveness of MDA-MB-231 cells was investigated using a Matrigel Invasion Chamber, as described previously^84^. In this assay, cancer cells must cross the matrigel matrix and the insert 8-µm pores (Costar, DC, USA). Cells (1 × 10^5^ cells in 0.5 ml of media with or without Ambrisentan) were seeded into the upper chamber of the Matrigel system. The bottom chamber in the system was filled with DMEM supplemented with 10% FBS as a chemo-attractant and then incubated at 37 °C for 24 h. Cells that have passed through the Matrigel and the pores were counted using the above described viability assay. The migration assay was performed using the chamber system without the matrigel matrix.

### Neutrophil viability assays

The effect of Ambrisentan on cell viability was analyzed by measuring neutrophil survival (apoptosis and necrosis) and function (respiratory burst and phagocytosis). After isolation of neutrophils as previously described^27^, the survival of neutrophils derived from healthy donors (all German subjects, age 22–45 years old) was analyzed using the Apoptotic/Necrotic/Healthy Cells Detection Kit (PromoCell, Heidelberg, Germany) according to the manufacturer´s instructions. The respiratory burst and phagocytosis capacities were assessed as previously described^80,85^. Briefly, 5 × 10^5^ neutrophils were cultured for 1 h at 37 °C in the absence or presence of phorbol-12-myristate-13-acetate (PMA, 300 ng/ml, Sigma-Aldrich, St. Louis, MO), followed by dihydrorhodamine (DHR 123, Merck KGaA, Darmstadt, Germany) staining. Neutrophils were stained with anti-CD15, and respiratory burst analyzed on a BD FACS Canto II Cytometer (BD Biosciences), gated according to size (forward scatter, FSC) and granularity (side scatter, SSC). Neutrophil phagocytic capacity was analyzed in whole blood by flow cytometry using *Staphylococcus aureus* BioParticles fluorescein conjugate (Thermo Fisher Scientific, Waltham, MA). After 1 h incubation at 37 °C, red blood cells were lysed using a red blood cell (RBC) lysis solution (Qiagen, Santa Clarita, CA) and phagocytosis was analyzed by flow cytometry and fluorescence microscopy (EVOS FL Cell Imaging System, Oakwood, OH, USA) as previously described^80^.

### Transwell migration assay

The inhibitory effects of Ambrisentan on neutrophil chemotaxis was analysed using the transwell migration assay (24-well plate, Corning, Inc., Corning, NY, USA) to analyse the response of non-adherent cells as previously described^27^. Samples of 5 × 10^5^ neutrophils or HL-60 cells were incubated before chemotaxis assays for 1 h in the absence or presence of 100 µM Ambrisentan. The cells were transferred to the upper chamber, while RPMI containing 10 nM n-formylmethionyl-leucyl-phenylalanine (fMLP) was placed in the lower chamber. The plate was incubated at 37 °C for 1 h. Cells that migrated towards the lower chamber of the transwell plates were transferred to a 96-well plate and counted (cells/µl) by flow cytometry using a CytoFLEX Flow Cytometer (Beckman Coulter, Indianapolis, IN, USA), gating on neutrophils according to FSC and SSC to exclude cell debris. Images of the cells on the bottom surface of the transwell plates were obtained using a fluorescence microscope (EVOS FL Cell Imaging System, Oakwood, OH, USA). The neutrophil migration capacity was calculated in relation to the spontaneous migration (cells with medium) which we arbitrarily considered as 100%.

### Oris migration assay

The migration of cancer adherent cell lines (COLO357 and OvCar3) was analyzed using the Oris migration assay^81^. COLO357 cells were cultured and allowed to migrate into the free space in the middle of the well for 48 h in the presence or absence of 100 µM PAR2 agonistic peptide (MoBiTec GmbH, Göttingen, Germany) and/or Ambrisentan. The cells were fixed and stained with a DiffQuick Cell Staining kit (Medion Diagnostics, Gräfelfing, Germany). After staining, an Oris detection mask was clipped to the bottom of the plate, and images were obtained with a blackfly camera on an Axioskop HBO 50 microscope (Zeiss, Oberkochen, Germany). The migration area was determined by analyzing the migration images with the Fiji module of the ImageJ software^86^. The pictures were analyzed with a pre-written macro^81^, analyzing each picture with the same thresholds for Grey values.

### RNA sequencing and bioinformatics analysis

RNA-seq was performed as previously described with minor modifications^80,87^. After culturing for 24 h in the presence or absence of 100 µM PAR2 agonistic peptide and/or 100 µM Ambrisentan, the total ribonucleic acid (RNA) from COLO357 cells was obtained using TRIZOL (Invitrogen, Carlsbad, CA, USA) according to the manufacturer’s instructions. RNA integrity and concentration were assessed using the Agilent 2100 Bioanalyser RNA Nano chip and orthogonally validated by visualization of the integrity of the 28S and 18S band on an agarose gel. Three independent experiments were performed and cells of each experimental condition pooled. cDNA libraries were obtained using the Illumina CBot station, and HiScanSQ using the NEBNext Ultra Sample Preparation Kit (Illumina Inc., San Diego, USA) according to each manufacturer’s instructions. Sequencing was carried out using the Illumina HiSeq 4000 platform (150-nucleotide paired-end reads). The bioinformatics analysis of data obtained was performed as previously described^88^. After quality assessment using FastQC^89^, reads were pseudo-aligned with the human cDNA transcriptome from the Ensembl 82 using Kallisto^90^ and differential gene expression was assessed using Sleuth^91^. The data obtained is available in Supplementary Table S1. A hierarchical clustering heatmap of all expressed genes was created using perseus (MaxQuant, v1.11, Martinsried, Germany)^92^ to show the Euclidian distance between the cell transcriptome under the different culture conditions.

### Mouse breast cancer model

BALB/c mice were purchased from Jackson Laboratory (Bar Harbor, ME, USA), bred in the animal facility at the College of Medicine and Health Science (CMHS, UAE University), housed in plastic cages with a controlled light and dark cycle of 12 h each and received rodent chow and water ad libitum. For orthotopic implantation, 4T1 cells (2 × 10^4^ cells in 100 µl volume, unless otherwise indicated) were injected subcutaneously into the fourth mammary fat pad of 8-week-old mice. Tumor growth was followed by quantitative determination of tumor volume twice weekly, as previously described^93^. Since Ambrisentan is poorly soluble in water, a working stock solution was prepared in dH_2_0 adjusted to a high pH (> 12). The stock solution was then re-adjusted to a physiological pH of ~ 7 with HCl to a final concentration of 20 mg/ml and kept at -20°c until use. Ambrisentan was diluted further to the desired concentration in ddH_2_0 before use. Before each experiment, the drug was further diluted to a working concentration of 1 mg/ml and was administered by oral gavage in a 200 µl volume per mouse (equivalent to ~ 10 mg/kg body weight). The experimental protocol involved random assignment of mice into two groups. Group I served as control and received a daily oral gavage of dH_2_0 for 5 days followed by a 2-day rest, for 2 consecutive weeks. Group II received Ambrisentan at 10 mg/kg body weight. At the beginning of week 3, all mice were implanted with 4T1 cells and treatment with Ambrisentan or dH_2_0 was continued for another 2 weeks post implantation. Mice were sacrificed at day 21 post-implantation and tumor, liver and lungs excised for further analysis. In separate experiments, the effect of Ambrisentan treatment on animal survival was assessed. Mice were implanted orthotopically with 4T1 cells (5 × 10^3^ cells per mouse) and tumor volume and animal survival were monitored for up to 60 days. Mice exhibiting signs of morbidity (weight loss, lethargy, piloerection, hyperkyphosis) were humanely sacrificed by CO_2_ asphyxiation.

### Lung tissue processing and flow cytometry

After the mice were sacrificed, the lungs were removed, washed with PBS, cut into small pieces using a scalpel, transferred into GentleMACS C-tubes (Miltenyi Biotec, Germany), and processed using the lung dissociation kit and a GentleMACS dissociator (Miltenyi), according to the manufacturer’s instructions. Homogenized lungs were passed through a 70-mm nylon mesh to obtain a single-cell suspension. The resultant cells were counted using an automated cell counter (EVE, NanoEnTek, Seoul, South Korea) and processed for flow cytometry, as detailed previously^94,95^. Briefly, cells were pre-incubated with anti-mouse CD16/32 mAb (Biolegend) to block FcγIII/II receptor sites and then stained with Zombie Aqua Viability dye (Biolegend). After washing, the cells were stained with a mixture of fluorochrome-conjugated antibodies using the following panel of mAbs: CD45-Alexa Fluor 700, CD11b-Alexa Fluor 488, Ly6G-BV605, Ly6C-APC-Cy7, F4/80-PE, and MHC II-BV785 (all from Biolegend). Data were collected on 30,000 cells per sample using FACS Celesta (BD) and analyzed using BD FACS Diva software (BD Bioscience).

### Assessment of liver metastasis

Liver tissues were fixed, embedded in paraffin and used to prepare thin sections (5 µm) using an established protocol^96^. Tissue sections were stained with hematoxylin and eosin and images captured with an Olympus BX51 microscope equipped with digital camera DP26 (Olympus Corporation, Tokyo, Japan). Quantification of micrometastases, defined as single-to small clusters of tumor cells, in the liver was done using H&E-stained sections and visualized in a stereo investigator system (Zeiss Imager M2 AX10, Germany). The total area of each section was scanned and measured. Digital images were used to quantify the number of tumor metastatic foci in each section and calculated as the number of foci per mm^2^ area. Indirect immunostaining was also used to detect highly proliferative cells with a rabbit anti-mouse Ki67 antibody (Abcam, Cambridge, UK) followed by anti-rabbit-HRP (Cell signaling, Danvers, MA, USA). Peroxidase activity was determined using DAB chromogen (Dako, Carpinteria, CA, USA). Sections were then counter-stained with hematoxylin and visualized and photographed with an Olympus BX51 microscope. All slides were examined by a certified pathologist under blinded conditions.

### Statistical analysis

Statistical significance was assessed by non-parametric Mann–Whitney tests. Data were expressed as median with range. For the animal studies, statistical significance between control and Ambrisentan-treated groups was analyzed by the unpaired two-tailed Student’s *t*-test. Survival analysis was performed by Kaplan–Meier survival curves and log-rank test (Mantel-Cox). The statistical analyses were performed using GraphPad PRISM 5.01 software (GraphPad Software, San Diego, CA), and differences with a *p* value ≤ 0.05 were considered significant.

### Ethics statement

All participants provided written informed consent. All experimental procedures were performed in accordance with the Declaration of Helsinki and approved by the ethics committees of the University of Lubeck. All mouse assays were conducted in accordance with, and after approval of the Animal Research Ethics Committee of the United Arab Emirates University.

## Supplementary information

Supplementary Figures.

Supplementary Table.

## Data Availability

The RNASeq data is provided as Supplementary Table S1. Additional data is provided upon reasonable request.
