# Supplementary material for: Ambrisentan, an endothelin receptor type A-selective antagonist, inhibits cancer cell migration, invasion, and metastasis
Source: Sci Rep. 2020 Sep 28;10:15931. doi: 10.1038/s41598-020-72960-1 (PMC7522204; doi:10.1038/s41598-020-72960-1)
Supplement: Supplementary file 1 — Supplementary Figures. [file 41598_2020_72960_MOESM1_ESM.docx]

**Supplementary Figures**

**Ambrisentan, an endothelin receptor type A-selective antagonist, inhibits cancer cell migration, invasion, and metastasis**

Lucy Kappes^a^**^*^**, Ruba L. Amer^b^**^*^**, Sabine Sommerlatte^a^, Ghada Bashir^b^, Corinna Plattfaut^c^, Frank Gieseler^c^, Timo Gemoll^d^, Hauke Busch^e^, Abeer Altahrawi^f^, Ashraf Al-Sbiei^b^, Shoja M. Haneefa^b^, Kholoud Arafat^g^, Lena F. Schimke^a^, Nadia El Khawanky^h^, Kai Schulze-Forster^i,j^, Harald Heidecke^i^, Anja Kerstein-Staehle^a^, Gabriele Marschner^a^, Silke Pitann^a^, Hans D. Ochs^k^, Antje Mueller^a^, Samir Attoub^g^, Maria J. Fernandez-Cabezudo^l^, Gabriela Riemekasten^a^, Basel K. al-Ramadi^b,#^ , Otavio Cabral-Marques^a,m,#^

**Supplementary Figure 1**

**A. B.**


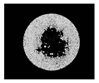

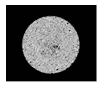

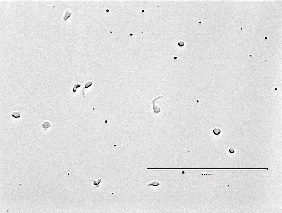

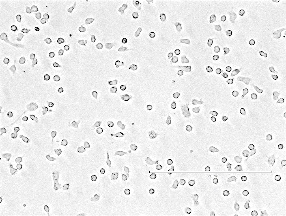


fMLP + Ambrisentan

Par2 ag + Ambrisentan

Par2 ag.

fMLP

**Ambrisentan blocks the migration of tumor cell lines.** (**A**) The graphic shows migration of ovarian carcinoma (OvCar3) cells, assessed as illustrated in figure 1a. Assays were performed in quadruplicates. Error bars denote mean with SD; *, p ≤ 0.05 (n = 3, Mann–Whitney test). While the spontaneous cell migration was inhibited by Ambrisentan (100µM) only a higher drug dose (500µM) was able to significantly block Par2- induced migration of OvCar3 cells (data not shown). Representative images (low panel) obtained using the Oris Pro Cell assay are shown. (**B**) The graphic displays % of migration of DMSO-pre-treated HL-60 in the presence or absence of Ambrisentan. Treatment of HL-60 cells for one hour in the presence of 100 µM Ambrisentan inhibited their movement toward fMLP. HL-60 migration was calculated in relation to the spontaneous migration (cells with medium only) which we arbitrarily considered as 100%. A representative image of three independent experiments using HL-60 cells that migrated toward fMLP on the bottom surface of transwell plates is shown (low panel). The results are representative of three independent experiments performed using transwell migration assays. Error bars denote mean with SEM; *, p ≤ 0.05 (n = 3; Mann–Whitney test).

**Supplementary Figure 2**

**Tumor cell lines express ETAR and ETBR.** Gene expression was performed by quantitative real-time PCR (qPCR) as described in supplementary material and methods.
